# A lipidated bi-epitope vaccine comprising of MHC-I and MHC-II binder peptides elicits protective CD4 T cell and CD8 T cell immunity against *Mycobacterium tuberculosis*

**DOI:** 10.1186/s12967-018-1653-x

**Published:** 2018-10-11

**Authors:** Pradeep K. Rai, Sathi Babu Chodisetti, Sudeep K. Maurya, Sajid Nadeem, Weiguang Zeng, Ashok K. Janmeja, David C. Jackson, Javed N. Agrewala

**Affiliations:** 1 CSIR-Institute of Microbial Technology, Chandigarh, India; 2 Department of Pulmonary Medicine, Government Medical College and Hospital, Chandigarh, India; 3 Department of Microbiology and Immunology, Peter Doherty Institute for Infection and Immunity, The University of Melbourne, Parkville, VIC 3010 Australia; 4 Indian Institute of Technology, Rupnagar, 140001 India; 5 Present Address: Department of Microbiology and Immunology, Pennsylvania State University College of Medicine, Hershey, PA 17033 USA

**Keywords:** Multistage vaccine, Promiscuous peptide, TLR-2, TB, *Mtb*, Th1 cells, Th17 cells

## Abstract

**Background:**

The clinical trials conducted at Chingleput India suggest that BCG fails to protect against tuberculosis (TB) in TB-endemic population. Recent studies advocate that non-tuberculous mycobacteria and latent *Mycobacterium tuberculosis* (*Mtb*) infection interferes in the antigen processing and presentation of BCG in inducing protective immunity against *Mtb.* Thereby, indicating that any vaccine that require extensive antigen processing may not be efficacious in TB-endemic zones. Recently, we have demonstrated that the vaccine candidate L91, which is composed of lipidated promiscuous MHC-II binder epitope, derived from latency associated Acr1 antigen of *Mtb* is immunogenic in the murine and Guinea pig models of TB and conferred better protection than BCG against *Mtb*.

**Methods:**

In this study, we have used a multi-stage based bi-epitope vaccine, namely L4.8, comprising of MHC-I and MHC-II binding peptides of active (TB10.4) and latent (Acr1) stages of *Mtb* antigens, respectively. These peptides were conjugated to the TLR-2 agonist Pam2Cys.

**Results:**

L4.8 significantly elicited both CD8 T cells and CD4 T cells immunity, as evidenced by increase in the enduring polyfunctional CD8 T cells and CD4 T cells. L4.8 efficiently declined *Mtb*-burden and protected animals better than BCG and L91, even at the late stage of *Mtb* infection.

**Conclusions:**

The BCG-L4.8 prime boost strategy imparts a better protection against TB than the BCG alone. This study emphatically denotes that L4.8 can be a promising future vaccine candidate for controlling active and latent TB.

**Electronic supplementary material:**

The online version of this article (10.1186/s12967-018-1653-x) contains supplementary material, which is available to authorized users.

## Background

Vaccine is the best prophylactic measure to prevent and control any disease. Currently, potent vaccines are unavailable for many dreaded diseases, including TB. TB is a major global threat among the leading killer diseases. It kills 1.8 million individuals annually, despite of the availability of effective drug regime [1]. The protective ability of the only available vaccine BCG against TB is highly controversial, since its efficiency is 0% in TB-endemic regions, whereas it is 80% in non-endemic zones [2, 3]. Therefore, high TB burden countries majorly contribute to the global TB cases. Further, BCG protection is restricted to childhood manifestation of the disease and its efficacy diminishes with age [2]. Similarly, it has been shown that many recombinant BCG or cell based vaccines have failed to protect against *Mtb* in TB-endemic population [4]. The major reasons suggested for the failure of BCG is due to the interference in the antigen processing and presentation by environmental mycobacteria and latent *Mtb* [2, 5–7]. This indicates that any vaccine that requires extensive antigen processing and presentation may not be quite successful in TB-endemic regions [2]. Hence, taking into consideration that immune landscape of TB-endemic population is quite distinct from non-endemic regions; a radical change is required while developing a global vaccine. Consequently, based on the above findings, peptide vaccine would be a perfect choice for TB-endemic residents [8]. Recently, we have synthesized a chimeric peptide L91, which comprises of a promiscuous CD4 T cell epitope of latently associated Acr1 antigen of *Mtb* and TLR-2 agonist Pam2Cys [9, 10]. The amino acid sequence 91–110 (F91) of the Acr1 protein was identified based on its permissive and high affinity binding (IC_50_ < 10 µM) to HLA class II alleles. Further, it chiefly elicited secretion of IFN-γ by T cells obtained from PPD^+^ healthy volunteers [11, 12]. The L91 stimulated DCs showed better cognate interaction and IFN-γ release by peptide specific CD4 T cells. Further, induction in the generation of enduring memory Th1 cells was observed. It is worth to mention here that L91 immunization protected mice and Guinea pigs better than BCG from *Mtb* [8]. Furthermore, L91 induced the proliferation of the PBMCs obtained from PPD^+^ healthy volunteers [8].

Besides CD4 T cells, CD8 T cells also considerably contribute in protecting against *Mtb* [13–15]. Hence, to enhance the efficacy of the vaccine, it is essential to incorporate both CD4 T cell and CD8 T cell epitopes. Further, *Mtb* persists in the host in distinct stages, namely active and latent. Therefore, for an improved vaccine, combination of multistage epitopes derived from active and latent form of disease is decisive. Henceforth, to achieve better protection, we improved our previous vaccine construct L91 by incorporating CD8 T cell epitope of the active form of *Mtb* antigen (TB10.4) (L4.8). In the current study, we observed that L4.8 efficiently activated both CD4 T cells and CD8 T cells and showed better protection than L91 and BCG vaccines. Thus, signifying that lipidated multistage epitopes based vaccine L4.8 may successfully confer protection against both the active and latent TB.

## Methods

### Animals

Female BALB/c mice (6–8 weeks) were procured from Animal Facility of the ‘Institute of Microbial Technology’. The animals were housed in individually ventilated cages and food and water was provided ad libitum.

### Vaccine constructs used in study

Lipidated peptides, free peptides and Pam2Cys used in the study were synthesized, as described elsewhere [9, 10]. The L91 vaccine construct consists of a promiscuous MHC-II binding peptide of a sequence SEFAYGSFVRTVSLPVGADE of 16 kDa α-crystalline protein of *Mtb* conjugated to Pam2Cys, a TLR-2 agonist. The lipidated multi-stage and multi-epitope vaccine for mouse experiments L4.8 comprises of SEFAYGSFVRTVSLPVGADE and BALB/c restricted MHC-I binding peptide of sequence GYAGTLQSL of TB10.4 antigen of *Mtb* [16] linked to Pam2Cys. The control non-mycobacterial lipidated peptide (LH) comprises of MHC-II and MHC-I binding peptides ALNNRFQIKGVELKS and TYQRTRALV, respectively of hemagglutinin protein of influenza virus coupled to Pam2Cys [9]. F91 and F4.8 signify non-lipidated form of L91 and L4.8, respectively (Additional file 1: Table S1).

### Immunization and infection

For short term immune response, mice were vaccinated subcutaneously (s.c.) at the base of tail with L4.8 (20 nmol) and 1 week later a booster was given with a half the dose (10 nmol) of peptide. One week later, animals were sacrificed, and cells isolated from spleen and lymph nodes were pooled. L4.8 specific T cell response was monitored following in vitro culturing with L4.8 (1 nmol), F4.8 (1 nmol), Pam2Cys (50 ng/ml) and medium alone for 72 h. For long-term immune response and protection studies, mice were immunized with a Danish strain of BCG (10^6^ CFU/animal). Twenty-one days later, mice were boosted twice with L4.8 and L91 (20 nmol and 10 nmol), at an interval of 2 weeks. Control groups were immunized with BCG alone, antigenically irrelevant lipidated hemagglutinin peptide (LH) of influenza virus and placebo (PBS). Ninety days after the last immunization, mice were aerosol challenged with *Mtb* (~ 100 CFU/animals) by aerosol machine Inhalation Exposure System (Glas-Col, LLC, Terre Haute, IN). After 30 days of infection, mice were sacrificed, and cells were isolated from the lungs, spleen and lymph nodes. L4.8 specific T cell response was examined following in vitro incubation with L4.8 (1 nmol), F4.8 (1 nmol), Pam2Cys (50 ng/ml) and medium alone for 72 h. For protection studies, animals were sacrificed after 90 days of infection and lungs and spleen were excised and homogenates were prepared. The homogenates were plated on 7H11 medium plate to monitor CFUs. Histopathological analysis was done after staining of fixed lung sections with hematoxylin and eosin. The results of L4.8 vaccine were compared with L91, BCG and Pam2cys or unless otherwise mentioned.

### Isolation of lymphocytes from lymph nodes, spleen and lungs

Spleens and lymph nodes obtained from immunized mice were pooled and a single cell suspension was prepared by gently pressing through frosted slides. The lungs were perfused using chilled PBS and small pieces were prepared and digested with collagenase (2 mg/ml) and DNase (0.03 mg/ml) for 30 min/37 °C. Later, cells were passed through a sieve (70 μM). Viability was checked by trypan blue exclusion method. The cells (2 × 10^5^/well) were added to 96 well U-bottom culture plates and incubated with L4.8 (1 nmol) and control cultures with F4.8 (1 nmol), L91 (1 nmol), Pam2Cys (50 ng/ml) and medium for 72 h/37 °C/5% CO_2_.

### In vivo CD8 T cells lytic activity

In vivo target lysis was determined by using standard protocol, described elsewhere [17]. Briefly, splenocytes were incubated with L4.8 (9 μM) at 37 °C/90 min. The control cells were incubated with medium alone, i.e. without L4.8. After incubation, cells were washed 2× with PBS. The L4.8 pulsed and un-pulsed cells were labeled with high (5 μM) and low (0.5 μM) concentrations of carboxyfluorescein succinimidyl ester (CFSE) dye respectively. After washing, cells were mixed in 1:1 ratio (3 × 10^7^ cells/100 μl PBS), and adoptively transferred in mice that were previously immunized twice with L4.8 or PBS at an interval of 1 week. After 16 h, mice were sacrificed, and single cell suspension of spleen cells was prepared. The percentage of cells expressing CFSE^hi^ and CFSE^lo^ was enumerated through flow cytometry. The following formula was used to calculate specific lysis: ratio = (mean percentage of CFSE^lo^ cells/mean percentage of CFSE^hi^ cells).

### Mycobacterial strains and BCG

H37Rv strain of *Mtb* was cultured in 7H9 medium containing Tween-80 (0.05%) supplemented with albumin (10%), dextrose and catalase (ADC). Glycerol stocks of H37Rv were prepared and stored at − 80 °C, and later used for infection studies. BCG vaccine (TUBERVAC) used for immunization was purchased from Serum Institute of India, Pune, India. TUBERVAC (Bacillus Calmette–Guerin vaccine I.P.) is a live freeze-dried vaccine derived from an attenuated strain of *Mycobacterium bovis* and meets the requirements of W.H.O. and I.P., when tested by the methods outlined in W.H.O., TRS. 745 (1987), 771 (1988) and I.P.

### Reagents and antibodies

Chemicals and reagents were purchased from Sigma Aldrich (St. Louis, MO). Anti–mouse flurochrome labeled antibodies (Abs): CD4-PB, CD8-APC-Cy7, CD62L-APC, CD44-PerCP-Cy5.5, CD127-PE, KLRG1-PE, IFN-γ-PECy7, TNFα-PerCPCy5.5, IL-17-PerCPCy5.5 and Abs for ELISA were procured from BD Pharmingen (San Diego, CA); CD27-FITC, CD43-PE, CXCR3-FITC and CCR6-APC from Biolegend (San Diego, CA) or otherwise mentioned. RPMI-1640 and FBS were purchased from GIBCO (Grand Island, NY). For culturing of cells, tissue culture grade plastic-ware was purchased from BD Biosciences (Bedford, MA).

### Proliferation assays

The cells (2 × 10^7^ cells) were incubated with CFSE dye (1 μM) in PBS (4 ml) at 37 °C. Free CFSE was quenched with 2 ml of FCS and excess was removed by washing 3× with RPMI-FCS-10%. CFSE-labeled cells were cultured with either L4.8 (1 nmol) or control cultures with F4.8 (1 nmol), L91 (1 nmol), Pam2Cys (50 ng/ml) and medium for 72 h. The proliferation of CFSE-labeled cells was analyzed by flow cytometry.

### Intracellular cytokine and surface staining

The cultures were set as mentioned in the proliferation assay. Later, these cultures were stimulated with PMA (50 ng/ml) and ionomycin (10 μM) for 2 h followed by incubation with brefeldin A (5 µg/ml) for additional 4 h. Cells were then harvested; washed 2× with buffer (PBS+FBS 2%) and fixed with paraformaldehyde (1%) at 4 °C/30 min. These cells were then perforated with saponin (0.2%) and incubated with fluorochrome tagged anti-IFN-γ, IL-17A and TNF-α Abs at 4 °C/90 min. The cells were washed with saponin (0.2%), followed by wash buffer. For surface staining of CD4, CD8, CD44, CD62L, CD127, CD27, CD43, KLRG1, CCR6 and CXCR3, cells were incubated with either fluorochrome labeled Abs or biotinylated Abs/streptavidin-fluorochrome conjugates. Standard protocols of washing/incubation were followed at each stage. Flow cytometry was carried out using FACS-Aria III and data were analyzed using BD FACS DIVA software package (BD Biosciences, San Jose, CA).

### Cytokine estimation by ELISA

The cultures were set as mentioned in T cell proliferation assay. After 72 h, culture SNs were harvested and cytokines were estimated by standard sandwich ELISA, as described by the manufacturer [18].

### Statistical analysis

The statistical analysis was performed employing ‘one-way ANOVA’ test for multiple comparisons.

## Results

### L4.8 elicits IFN-γ and IL-17 secreting CD4 T cells and CD8 T cells

**Fig. 1:**
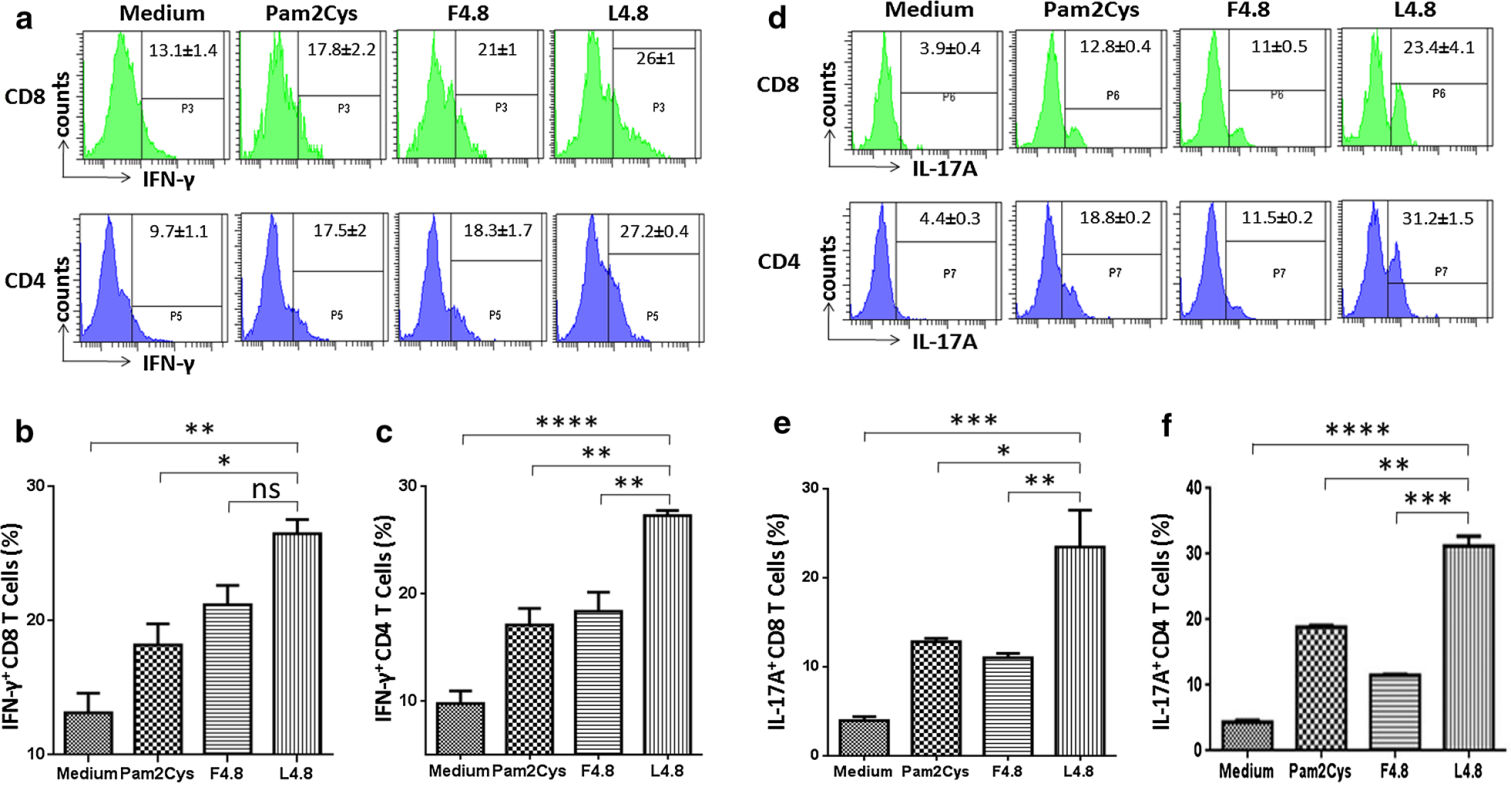
L4.8 elicits secretion of IFN-γ and IL-17 by both CD8 T cells and CD4 T cells. Mice were immunized twice with L4.8 (20 nmol) at an interval of 1 week. After 1 week, animals were sacrificed and cells from the spleen and lymph nodes were pooled and single cell suspension was prepared. The cultures were set and stimulated with L4.8. Control cultures were incubated with F4.8 and Pam2Cys for 72 h. Later, CD8 and CD4 gated T cells were analyzed for the intracellular expression of IFN-γ and IL-17A. **a** Flow cytometry histogram and **b**, **c** bar diagrams indicate IFN-γ expressing CD8 T cells and CD4 T cells. Likewise, **d** histogram and **e**, **f** bar diagrams represent IL-17A expressing CD8 T cells and CD4 T cells. Data represented as mean ± SEM are percent population from 2 independent experiments (n = 3 mice/group). *p ≤ 0.05, **p ≤ 0.005, ***p ≤ 0.0005, ****p ≤ 0.0001

We evaluated immunogenicity of L4.8 vaccine construct for the elicitation of CD4 T cells and CD8 T cells immunity. BALB/c mice were s.c. vaccinated twice at the base of tail with L4.8 at an interval of 7 days with 20 nmol and 10 nmol, respectively. Mice were sacrificed 7 days after the booster dose and single cell suspensions were prepared from spleen and inguinal lymph nodes. Thereafter, the cells were in vitro stimulated with L4.8 (optimal titrated dose, 1 nmol, Additional file 2: Figure S1B) and control cultures with F4.8, Pam2Cys and medium. The intracellular expression of IFN-γ and IL-17A was monitored on CD4 and CD8 gated T cells (Additional file 2: Figure S2). It was observed that L4.8 stimulation elicited strong Th1, Th17, Tc1 and Tc17 immune response, as evidenced by significantly higher intra cellular expression of IFN-γ (CD8: *p *≤ 0.05; CD4: *p *≤ 0.005) (Fig. 1a–c) and IL-17 (CD8: *p *≤ 0.05; CD4: *p *≤ 0.005) by CD8 T cells and CD4 T cells, as compared to Pam2Cys (Fig. 1d–f).

### L4.8 induces better IFN-γ secretion than L91

**Fig. 2:**
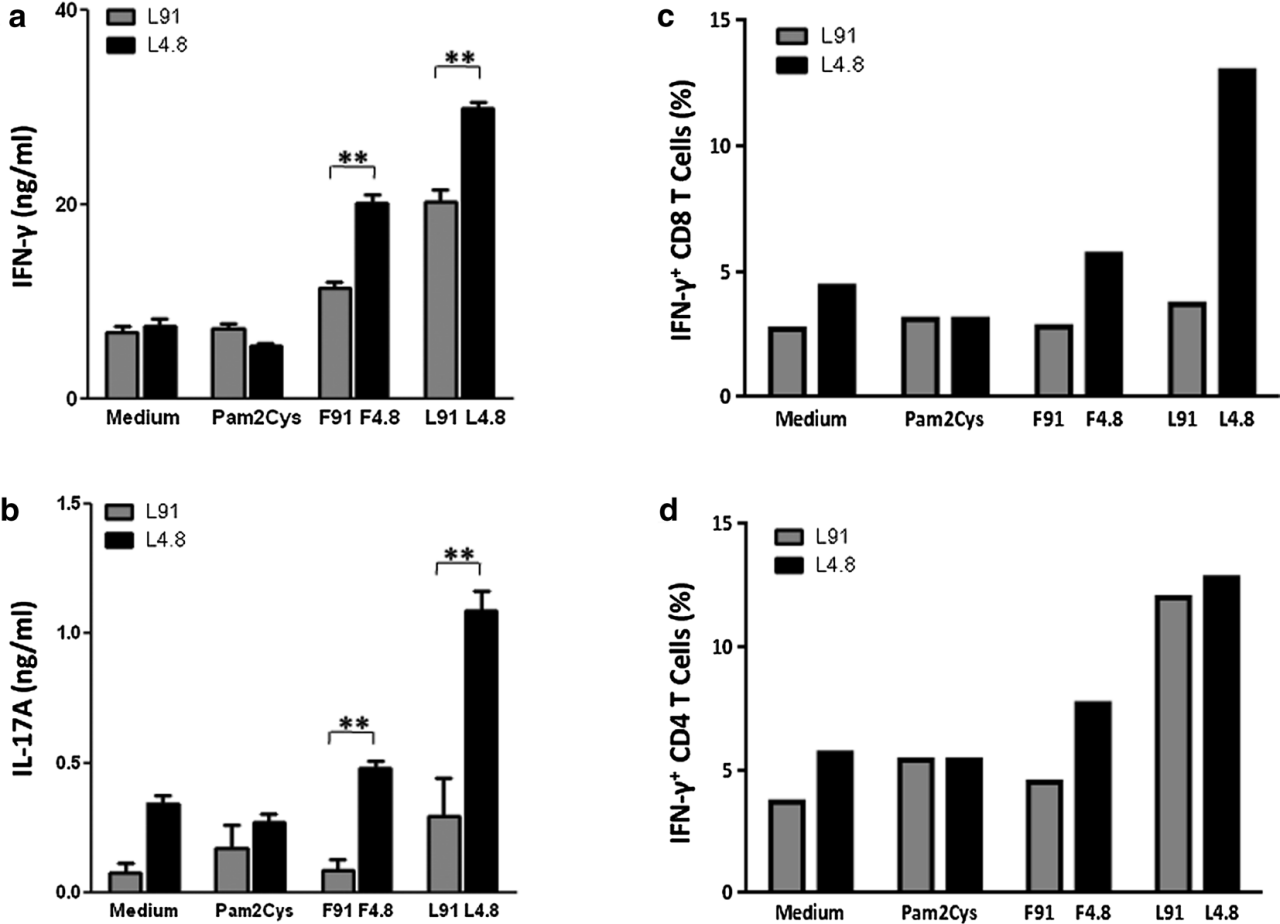
Induction of the protective immune response by L91 and L4.8. The cultures were set as mentioned in the legend to Fig. 1. For L91 group, mice were immunized twice with L91 (20 nmol) at an interval of 1 week. Single cell suspension was prepared from the spleens and lymph nodes obtained from L91 and L4.8 immunized mice. **a** IFN-γ and **b** IL-17 were estimated in the culture SNs by ELISA and expressed as ng/ml. **c**, **d** Intracellular expression of IFN-γ was assessed by flow cytometry on **c** CD8 and **d** CD4 gated T cells. Results are presented as mean ± SEM, (n = 3 mice/group). **a**–**b** data are representative of 2 independent experiments; **c**, **d** pooled splenocytes of three mice from single experiment. **p ≤ 0.005. L91: mice immunized with L91; L4.8: mice immunized with L4.8

L91 is known to activate CD4 T cells to secrete mainly IFN-γ [8]. Hence, we compared the potency of L4.8 with L91 in inducing the production of IFN-γ. We compared the antigen specific recall response between L91 and L4.8 immunized animals with equimolar concentration of these peptides. We observed that cell suspension obtained from L4.8 immunized animals induced higher yield of IFN-γ (*p *≤ 0.005) and IL-17A (*p *≤ 0.005) than L91 during recall response (Fig. 2a, b). The intracellular expression of IFN-γ by CD8 T cells and CD4 T cells was monitored by flow cytometry. It was noted that L4.8 elicited higher pool of IFN-γ^+^ CD8 T cells, as compared to L91. No noticeable difference was observed in the percentage of IFN-γ^+^ CD4 T cells stimulated with either L91 or L4.8 (optimal titrated dose, 1 nmol, Additional file 2: Figure S1A, B) (Fig. 2c, d). These results suggest that since L4.8 comprises of both CD4 T cells and CD8 T cells epitopes, hence L4.8 induced secretion of IFN-γ by both CD4 T cells and CD8 T cells, while L91 induced the production of IFN-γ by only CD4 T cells.

### L4.8 immunization evokes CTL response

**Fig. 3:**
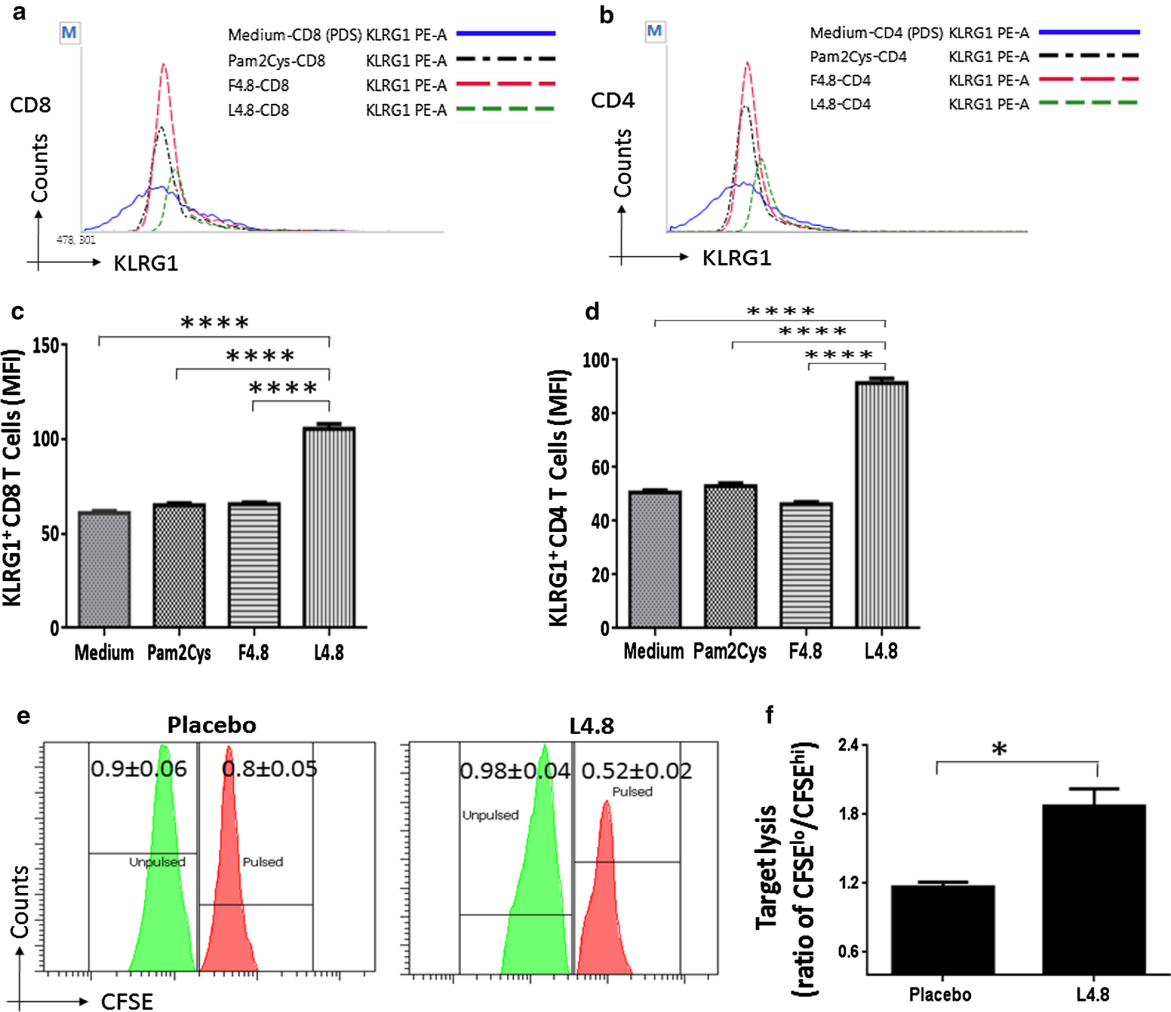
Immunization with L4.8 evokes CTL activity. Mice were immunized, and cultures were set as mentioned in the legends to Fig. 1. The expression of KLRG1 was monitored on CD4 T cells and CD8 T cells by flow cytometry and represented as: **a**, **b** histogram plots; **c**, **d** bar diagrams. **e**, **f** The lytic ability of CTL was evaluated by in vivo killer T cells assay. The data expressed as target lysis are calculated from the ratio of epitope-pulsed CFSE^hi^ and un-pulsed CFSE^lo^ to targets. The results are depicted as mean ± SEM (n = 3 mice/group). *p < 0.05, ****p ≤ 0.0001

CD8 T cells mediated target lysis is a well-established protective response during intracellular infections [9]. CD4 T cells also exhibit killer activity [18]. KLRG1 is a well-established marker associated with the activated killer T cells [19, 20]. Intriguingly, we observed that both CD8 T cells (*p *≤ 0.0001) and CD4 T cells (*p *≤ 0.0001) obtained from L4.8 immunized animals displayed considerable enhancement in the expression of KLRG1, when in vitro challenged with L4.8; as compared to control cells incubated with either F4.8 or Pam2Cys or medium (Fig. 3a–d). It is worth to mention here that the expression of KLRG1 was evaluated on CD8 and CD4 gated T cells (Additional file 2: Figure S3A). Further, we also observed significantly (*p *≤ 0.05) higher in vivo target lysing capability of T cells isolated from L4.8 immunized animals, as compared to control placebo group (Fig. 3e, f and Additional file 2: Figure S3B). These results indicate that L4.8 has enough potential to elicit cells adept of lysing targets.

### L4.8 elicits long-term polyfunctional CD8 T cells and CD4 T cells

**Fig. 4:**
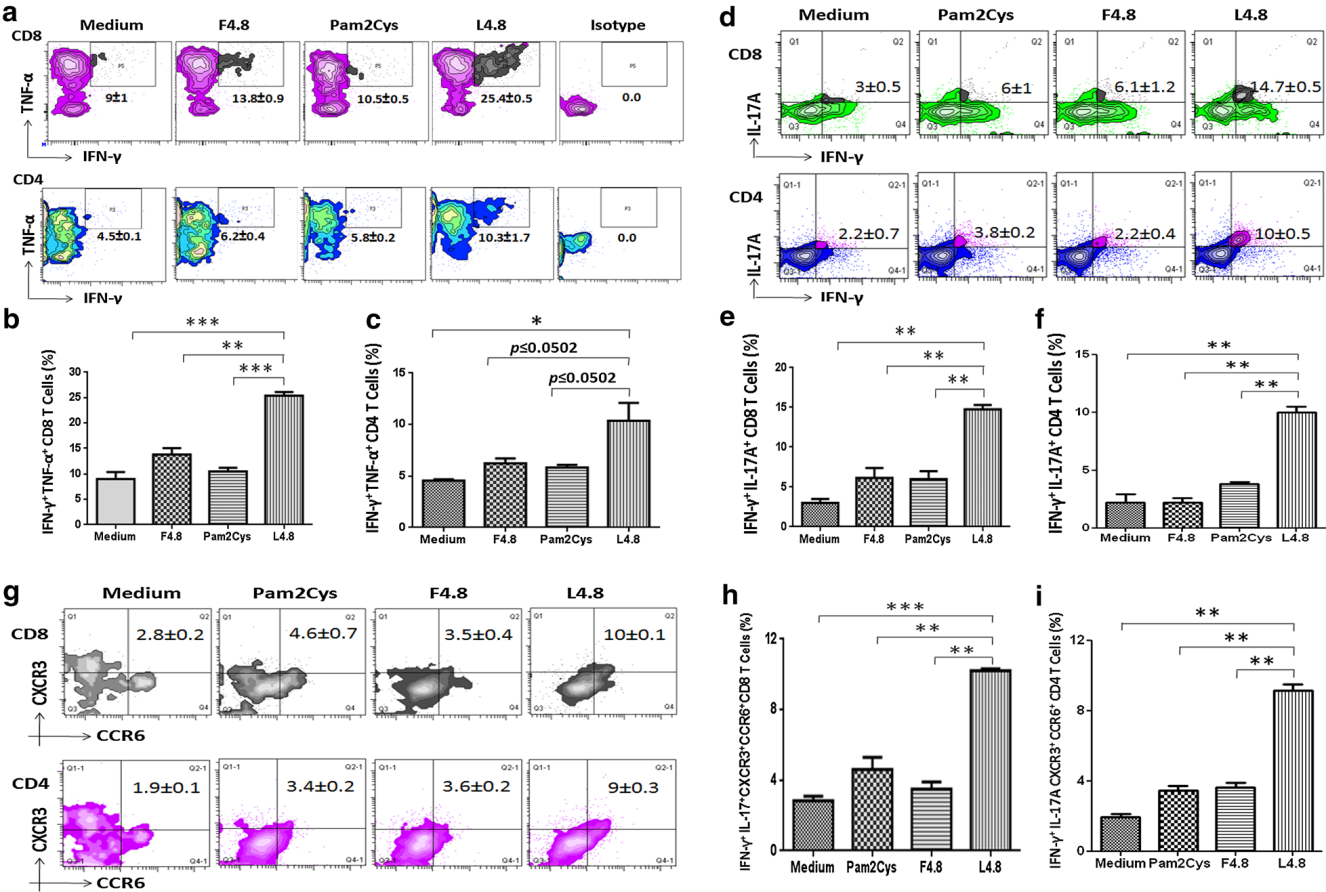
L4.8 immunization augments the pool of polyfunctional CD4 T cells and CD8 T cells. Single cell suspension was prepared from spleen of vaccinated mice and stimulated with L4.8. The polyfunctional CD8 T cells and CD4 T cells were monitored for the intracellular expression of cytokines by flow cytometry. **a** Contour plots and their **b**, **c** bar diagrams represent percent population of cells co-expressing IFN-γ and TNF-α. Similarly, **d** contour plots and their **e**, **f** bar diagrams correspond to cells co-expressing IFN-γ and IL-17A. IFN-γ^+^/IL-17A^+^ CD8 T cells and CD4 T cells were analyzed for the co-expression of CXCR3 and CCR6. **g** Contour plots and their **h**, **i** bar diagrams represent percent population of IFN-γ^+^/IL-17A^+^/CXCR3^+^/CCR6^+^ CD4 T cells and CD8 T cells. Data (mean ± SEM) represented as percent positive cells and are of 2 independent experiments (n = 3 mice/group). *p ≤ 0.05, **p ≤ 0.005, ***p ≤ 0.0005

The polyfunctional T cells show better protective efficacy compared to their counterparts secreting single cytokine since they express multiple cytokines [21–23]. After favorable results obtained from the short-term experiments with L4.8, we next examined its long-term protective efficacy against *Mtb*. In this case, we adopted BCG-L4.8 prime boost model. Mice were immunized with BCG and after 21 days, vaccinated with two doses of L4.8 at 14 days of interval. One of the cardinal features of any vaccine is to induce long-term immune response. Consequently, after final vaccination we rested the mice for 90 days to generate bonafide memory T cells, and later infected with *Mtb*. After 30 days, animals were sacrificed and single cell suspension from lungs was prepared and in vitro incubated with L4.8 and control cultures with F4.8, Pam2Cys and medium. The intracellular expression of cytokines was monitored on CD4 and CD8 gated T cells (Additional file 2: Figure S4A–D, Fig. 4a–c). The L4.8 stimulation generated substantially higher percentage of polyfunctional CD4 T cells and CD8 T cells co-expressing IFN-γ/TNF-α (CD8: *p *≤ 0.0005; CD4: *p *≤ 0.0502) (Fig. 4a–c) and IL-17A/IFN-γ (CD8: *p *≤ 0.005; CD4: *p *≤ 0.005) (Fig. 4d–f), as compared to controls. In contrast, control group vaccinated with BCG showed lesser frequency of IFN-γ^+^/TNF-α^+^ T cells as compared to BCG-L4.8 group and no change was observed with isotype-matched controls (Additional file 2: Figure S4A, B). The secretion of IFN-γ with TNF-α by T cells has a direct correlation with protection against *Mtb* by inducing autophagy and apoptosis of infected cells [24, 25]. Further, role of IL-17A has been explored to impart protection by chemokine-mediated recruitment of Th1 cells at the site of infection [26, 27]. The Th17 cells secreting IL-17A in conjunction with IFN-γ are known to show better protection against *Mtb*, due to the expression of CXCR3 and CCR6 [27]. Therefore, we next checked the expression of CXCR3 and CCR6 on IL-17A^+^ and IFN-γ^+^ cells (Fig. 4d). We observed that IL-17A^+^ and IFN-γ^+^ co-expressing cells exhibited double positive phenotype of CXCR3 and CCR6 (CD8: *p* ≤ 0.005; CD4: *p* ≤ 0.005) upon L4.8 stimulation (Fig. 4g–i). Although, the CD4 T cells and CD8 T cells that are expressing IL-17, IFN-γ, CXCR3 and CCR6 is a small population but their role in protection will be quite crucial due to their migratory property and production of cytokines that are important in imparting protection against *Mtb*.

### L4.8 vaccination augments enduring memory CD4 T cells and CD8 T cells

**Fig. 5:**
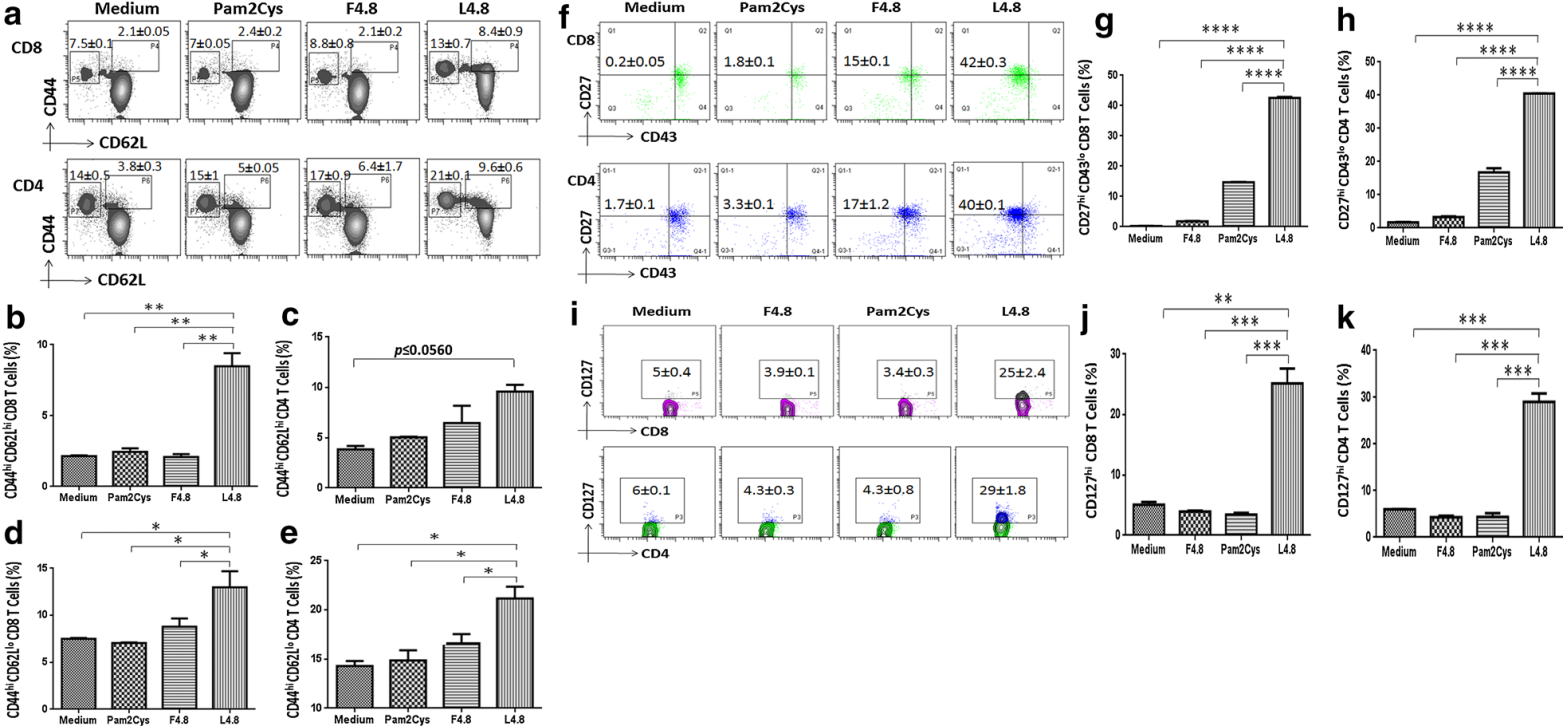
L4.8 immunization generates long-lasting memory CD4 T cells and CD8 T cells against *Mtb*. Mice were immunized and splenocytes culture was set as mentioned in the legends to Fig. 4. **a** Contour plots and their **b**–**e** bar diagrams indicate percentage of CD4 and CD8 gated T cells population expressing central memory (CD62L^hi^CD44^hi^) and effector memory (CD62L^lo^CD44^hi^) phenotype. **f** Dot plots and their **g**, **h** bar diagrams represent percentage population of CD27^hi^CD43^lo^ CD4 T cells and CD8 T cells. Likewise, **i** contour plots and **j**, **k** bar diagrams indicate percentage of CD4 and CD8 gated T cells (Additional file 2: Fig. S4A) expressing CD127^hi^ population. The data (mean ± SEM) in the inset are percent positive cells and representative of 2 independent experiments (n = 3 mice/group). *p ≤ 0.05, **p ≤ 0.005, ***p ≤ 0.0005, ****p ≤ 0.0001

The vaccinated mice were rested for 90 days, and later exposed to *Mtb* and subsequently sacrificed after 30 days. A single cell suspension was prepared from the spleen and incubated with L4.8, F4.8 and Pam2Cys. We observed substantial expansion in the pool of central (CD62L^hi^CD44^hi^) (CD8: *p *≤ 0.005; CD4: *p *≤ 0.0560) and effector memory (CD62L^lo^CD44^hi^) (CD8: *p *≤ 0.05; CD4: *p *≤ 0.05) CD8 T cells and CD4 T cells in a group that was vaccinated with L4.8 (Fig. 5a–e). These data were further corroborated by observing the significant expansion of CD27^hi^CD43^lo^ (CD8: *p *≤ 0.0001; CD4: *p *≤ 0.0001) and CD127^+^ (IL-7R) (CD8: *p *≤ 0.0005; CD4: *p *≤ 0.0005) memory population (Fig. 5f–k). The CD4 and CD8 gating strategy was followed as mentioned in Additional file 2: Figure S4A. IL-7R signaling is essential for the persistence of memory T cells [28–30]. In addition, T cells expressing CD27^hi^CD43^lo^ are responsible for maintaining enduring memory T cells and rapid recall response [31, 32]. These results signify the unique ability of L4.8 in inducing the generation and maintenance of long-term memory T cell response.

### L4.8 booster in BCG primed animals confers better protection than BCG alone

**Fig. 6:**
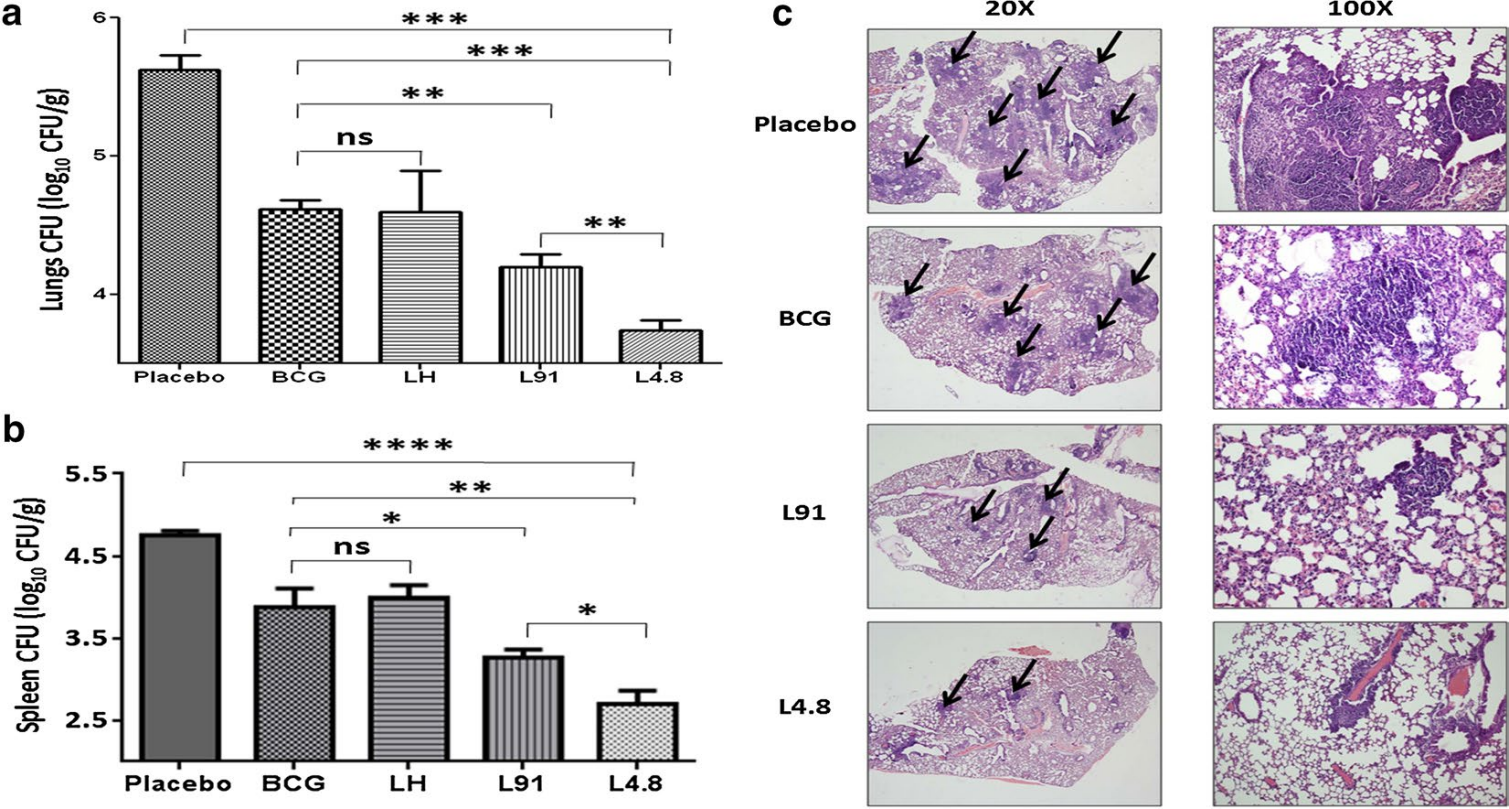
L4.8 vaccination protects from *Mtb*. BCG primed mice were boosted after 21 days twice with L4.8 and control groups with L91 or lipidated hemagglutinin virus peptide (20 nmol) or with PBS at an interval of 2 weeks. After 90 days, mice were infected with *Mtb*. Ninety days later, bacterial burden in the lungs was enumerated by CFUs. Bar diagram represents CFUs per gram of **a** lung and **b** spleen tissue. **c** Histopathological section of lungs were stained with hematoxylin and eosin and examined at a magnification ×20/×100. The arrows indicate granulomas or tubercle. Experimental groups signify that mice inoculated with ‘Placebo’: PBS, BCG: BCG, LH: BCG priming and LH (hemagglutinin peptide) boosting, L91: BCG priming and L91 boosting, L4.8: BCG priming and L4.8 boosting. Results shown as bar diagram are mean ± SEM and pooled data from 2 independent experiments (n = 3 mice/group). *p ≤ 0.05, **p ≤ 0.005, ***p ≤ 0.0005, ****p ≤ 0.0001

Next, we authenticated the protective efficacy of the L4.8 in BCG primed animals by monitoring the bacterial burden in the lungs and spleen of the vaccinated mice. Mice were aerosol challenged with *Mtb* after 90 days of last immunization. Later, bacterial burden were enumerated in the lungs and spleen after 90 days of infection. L4.8 vaccinated animals showed significant reduction in the bacterial counts, as compared to BCG (*p *≤ 0.0005) or L91 (*p *≤ 0.005) or placebo (*p *≤ 0.0005) (Fig. 6a). Further, L4.8 efficiently restricted the dissemination of bacilli, as compared to BCG (*p *≤ 0.005) and L91 (*p *≤ 0.05) vaccinated animals, as evidenced by significant decrease in the CFUs in spleen (Fig. 6b). Furthermore, histopathological analysis of infected lungs of L4.8 immunized mice supports the CFU data. The lungs showed lesser number of granulomas, restricted infiltration of lymphocytes and histiocytes, preserved alveolar spaces and normal architecture, compared to BCG. On the contrary, lungs of control mice administered with PBS exhibited more number of granulomas and larger confluent areas of consolidated lymphocytes and histiocytes (Fig. 6c). In agreement with the above observations, multi-epitope vaccine construct L4.8 imparts better protection than BCG and single-epitope based vaccine L91 and hence, can be a prudent choice of future vaccine against TB.

## Discussion

The fact remains that more people have been vaccinated with BCG than any other vaccine, yet TB continues to inflict scourge by killing 1.8 million people and infecting 10 million individuals annually [1]. Further, the anguish is compounded by the emergence of drug resistant strains of *Mtb* and AIDS pandemic [2]. Paradoxically, the efficiency of only available vaccine against TB BCG is considerably paralyzed in TB-endemic regions [3, 7, 33]. Its efficacy fades with time, which is evident by the increase in the number of TB cases with age [34]. Consequently, elucidating the fact that BCG fails to induce enduring immune memory [3]. BCG does not work adequately in helminth-infested individuals [7, 35]. In addition, BCG induces Th2 cells and regulatory T cells, which suppress immunity against *Mtb* [2]. Furthermore, several studies performed recently reveal that the inefficiency of BCG is because of impairment in the antigen processing and presentation by antigen presenting cells (APCs), which is caused by NTM and latent *Mtb* [2, 36–39]. Thus, making control of TB a daunting task in TB-endemic countries. Hence, any vaccine that does not require extensive antigen processing may work for TB-endemic population.

Recently, we have demonstrated that promiscuous peptide of *Mtb* conjugated to TLR-2 agonist Pam2Cys (L91), activated both innate and adaptive arms of immunity [8]. L91 bolstered the efficacy of dendritic cells (DCs) by increasing the yield of IL-6 and IL-12, upregulating the expression of costimulatory molecules and enhanced their ability to activate T cells. TLR-2 is copiously expressed on DCs [40]. Thus, L91 can be targeted to these highly professional APCs. DCs are the only cells in the entire immune system that have capability to active naive T cells [41–43]. Further, L91 induced long-term protection in mice and Guinea pigs by generating enduring memory Th1 cells and the response was considerably better than BCG. Consequently, signifying that lipidated peptides will work as successful vaccines, where the conventional ones have failed to protect. L91 is composed of only CD4 T cell epitope derived from latent stage antigen (Acr1) of *Mtb*; we further introduced CD8 T cell epitope from an early stage antigen TB10.4 in the novel construct L4.8 to improve the efficacy of the vaccine. Besides CD4 T cells, CD8 T cells also play a crucial role in imparting protection against *Mtb* [44]. Therefore, L4.8 may show a better protection than L91. In addition, its epitopes are well defined, and since it is a synthetic vaccine, there will be no concern of infection. Furthermore, it can induce long-lasting memory T cells [8, 45]. The only snags we perceive currently with L4.8 are its cost effectiveness and synthesis for mass immunization.

To monitor the efficacy of the construct, mice were vaccinated with L4.8 and following major findings emerged from the study regarding CD4 T cells and CD8 T cells: (i) substantial activation of both the subtype of cells; (ii) increase in the frequency of IFN-γ and IL-17A producing cells; (iii) robust expansion in the pool of multifunctional T cells, producing a combination of IFN-γ/TNF-α and IL-17A/IFN-γ cytokines; (iv) augmentation in the percentage of central and effector memory response; (v) elicitation of cytotoxic T cells; (vi) significant reduction in the bacterial burden in the lungs and spleen and thereby protection against *Mtb*.

The most striking findings of the study were that L4.8 significantly declined the *Mtb* burden in mice compared to BCG and L91, establishing the superiority of L4.8 over these vaccines. It is worth to mention here that the animals were rested for 180 days after vaccination, thus indicating L4.8 elicited long-lasting immunological memory response. The generation of the enduring immunity is a fundamental feature of any successful vaccine [46]. Unfortunately, BCG only protects children but not adults from TB. Further, it was observed that several vaccines that were recently undergoing clinical trials failed to engender persistent memory response [47, 48]. However, in the case of L4.8, considerable increase in the pool of persistent central and effector memory CD4 T cells and CD8 T cells was noticed. Further, we noticed qualitative long-term Th1, Th17, Tc1 and Tc17 immunity. Both Th1 cells and Th17 cells play a cardinal role in protecting against *Mtb* [49, 50]. Furthermore, we observed that L4.8 predominantly promoted polyfunctional CD4 T cells and CD8 T cells with concomitant expression of ‘IFN-γ with TNF-α’ and ‘IL-17A with IFN-γ’. The polyfunctional T cells producing multiple cytokines are qualitatively better than their counterparts secreting single cytokine [22, 51]. Consequently, these results illustrates that L4.8 generates immune response that plays a cardinal role in protection against *Mtb*.

Regrettably, infants that are immunocompromised or infected with HIV cannot be vaccinated with BCG, since they are highly vulnerable to BCG dissemination. Thus, leaving a choice for search of an appropriate vaccine for this population [52–54]. Lipidated peptide construct is totally synthetic and therefore can be a future hope for protecting not only healthy individuals but also immuno-compromised subjects and children suffering from AIDS.

## Conclusion

Currently, there are many whole cell-based vaccines including recombinant BCG, attenuated *Mtb*, etc. ready for clinical trials. However, they may encounter same problem of interference of non-tuberculous mycobacteria and helminth infestation in TB-endemic regions, as was associated with BCG failure. Consequently, peptide vaccines can overcome the aforesaid hurdles and may be a future choice of prophylactic measure to control TB.

## Additional files

**Additional file 1: Table S1.** Description of lipidated and free peptides in the vaccine. Table shows the detail of the sequence of MHC class I and MHC class II binding promiscuous peptides and the presence or absence of Pam2Cys used in the vaccine constructs. mL4.8/mF4.8 are murine restricted epitopes. ‘+’: presence; ‘−’: absence.

**Additional file 2: Figure S1.** Titration of peptide dose for in vitro stimulation. Mice were immunized with L91 or L4.8 (20 nmol) and boosted after 7 d (10 nmol). Splenocytes were obtained after 15 d of immunization and in vitro stimulated with different concentrations of (A) L91 and (B) L4.8 for 72 h. Splenocyte proliferation was assessed by ^3^H-thymidine incorporation assay and expressed as counts per minute (cpm). **Figure S2.** Gating strategy for CD4^+^ T cells and CD8^+^ T cells. The P1 gate was made on lymphocytes, P2 gate on SSC-A/CD4^+^ T cells and P3 on SSC-A/CD8^+^ T cells zones. Further, analysis of T cells was done on the gated cells. **Figure S3.** Gating strategy for monitoring the expression of KLRG1^+^ T cells and target lysis. (A) The P1 gate was made on lymphocyte zone. P2 gate on SSC-A and CD4^+^ T cells while P3 gate on SSC-A and CD8^+^ T cells. (B) For target lysis two gates were made in CFSE^hi^ and CFSE^lo^ cells on SSC-A. Later, histogram was plotted for the analysis. **Figure S4.** Gating strategy for monitoring the expression of IFN-γ^+^ and TNF-α^+^ CD4 T cells. (A, B) The primary gate was made on lymphocyte zone and secondary gate on SSC-A and (A) CD4^+^ T cells; (B) CD8 T cells. The percentage of IFN-γ^+^/TNF-α^+^ was monitored on secondary zones (CD4^+^/SSC-A; CD8^+^/SSC-A). The isotype-matched control did not show any double positive T cell population. (C, D) Bar diagram represents the comparison of polyfunctional (C) CD4 T cells and (D) CD8 T cells (IFN-γ^+^ TNF-α^+^) obtained from BCG and BCG-L4.8 groups. The data are represented as mean ± SEM. *p ≤ 0.05; ****p ≤ 0.0001.

## Abbreviations

TB: tuberculosis
*Mtb*: *Mycobacterium tuberculosis*
BCG: Bacillus Calmette–Guerin
IFN: interferon
TNF: tumor necrosis factor
IL: interleukin
CD: cluster of differentiation
TLR: toll like receptor
Acr: alpha-crystallin
PBS: phosphate buffered saline
CFU: colony forming unit
CFSE: carboxyfluorescein succinimidyl ester
PMA: phorbol myristate acetate
CCR/CXCR: chemokine and cytokine receptor
MHC: major histocompatibility complex
