# Supplementary material for: A lipidated bi-epitope vaccine comprising of MHC-I and MHC-II binder peptides elicits protective CD4 T cell and CD8 T cell immunity against Mycobacterium tuberculosis
Source: J Transl Med. 2018 Oct 11;16:279. doi: 10.1186/s12967-018-1653-x (PMC6180631; doi:10.1186/s12967-018-1653-x)
Supplement: Supplementary file 1 — Additional file 1: Table S1. Description of lipidated and free peptides in the vaccine. Table shows the detail of the sequence of MHC class I and MHC class II binding promiscuous peptides and the presence or absence of Pam2Cys used in the vaccine constructs. mL4.8/mF4.8 are murine restricted epitopes. ‘+’: presence; ‘−’: absence. [file 12967_2018_1653_MOESM1_ESM.docx]

**Supplementary Table 1**

| **Vaccine construct** | **Pam2Cys** | **MHC-I binder epitope** | **MHC-II binder epitope** |
| --- | --- | --- | --- |
| L91 | + | - | SEFAYGSFVRTVSLPVGADE |
| F91 | - | - | SEFAYGSFVRTVSLPVGADE |
| mL4.8 | + | GYAGTLQSL | SEFAYGSFVRTVSLPVGADE |
| mF4.8 | - | GYAGTLQSL | SEFAYGSFVRTVSLPVGADE |
| LH | + | TYQRTRALV | ALNNRFQIKGVELKS |
