# Supplementary material for: A lipidated bi-epitope vaccine comprising of MHC-I and MHC-II binder peptides elicits protective CD4 T cell and CD8 T cell immunity against Mycobacterium tuberculosis
Source: J Transl Med. 2018 Oct 11;16:279. doi: 10.1186/s12967-018-1653-x (PMC6180631; doi:10.1186/s12967-018-1653-x)

Supplementary Figure 1A: Dose titration of peptides for *in-vitro* stimulation

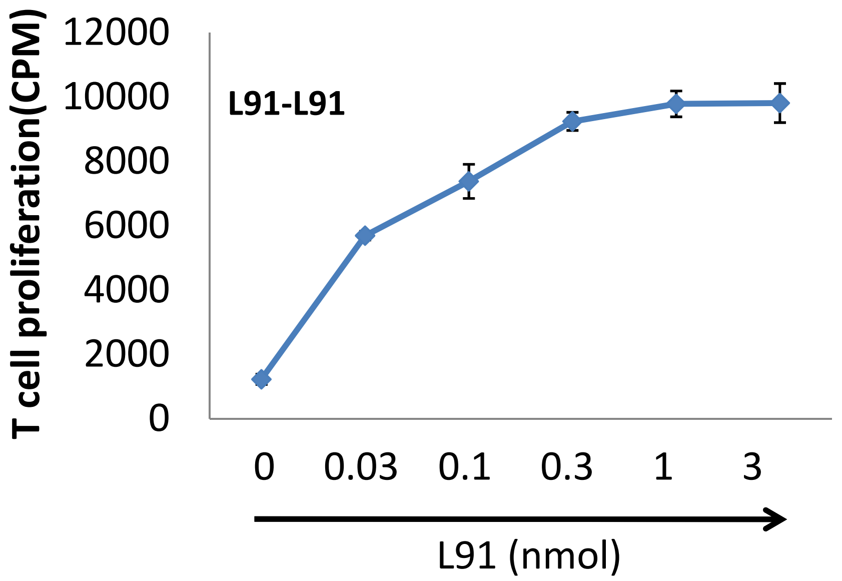

Supplementary Figure 1B

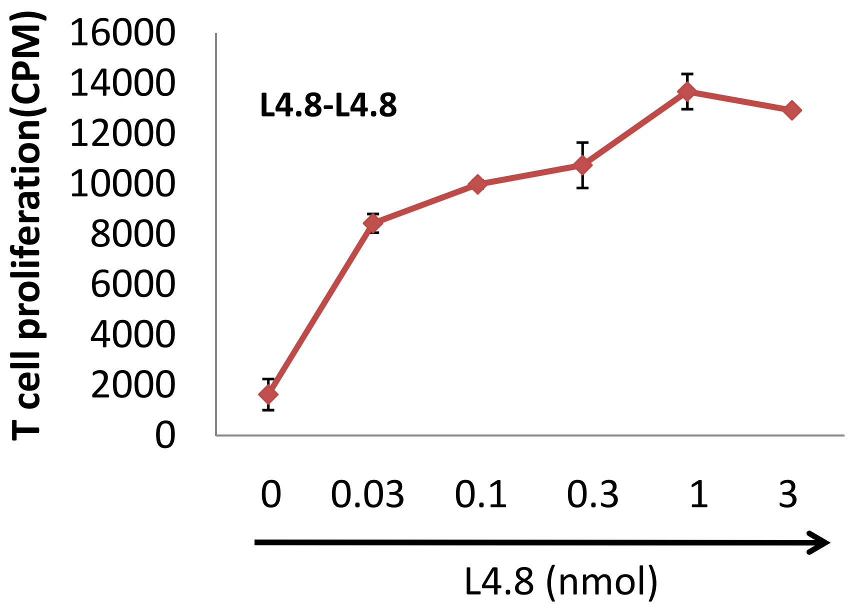

Supplementary Figure 2

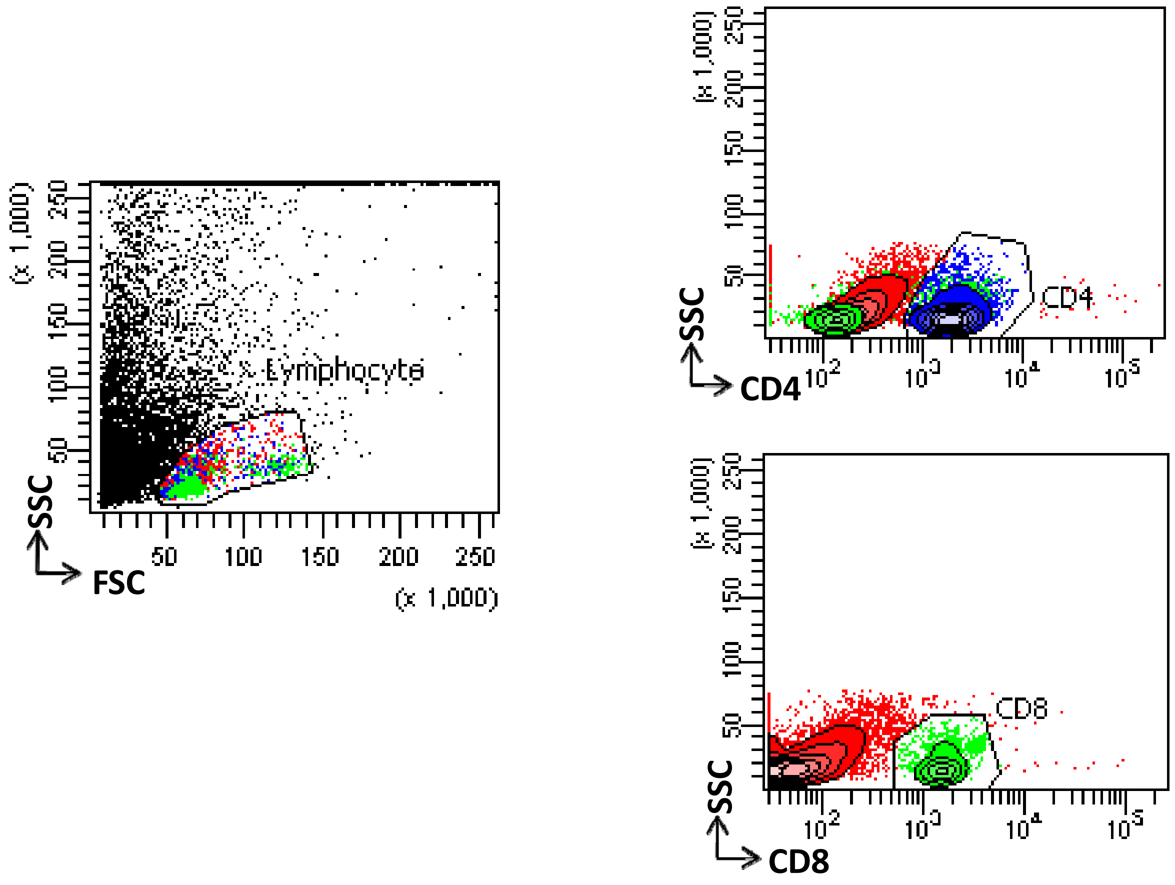

Supplementary Figure 3A

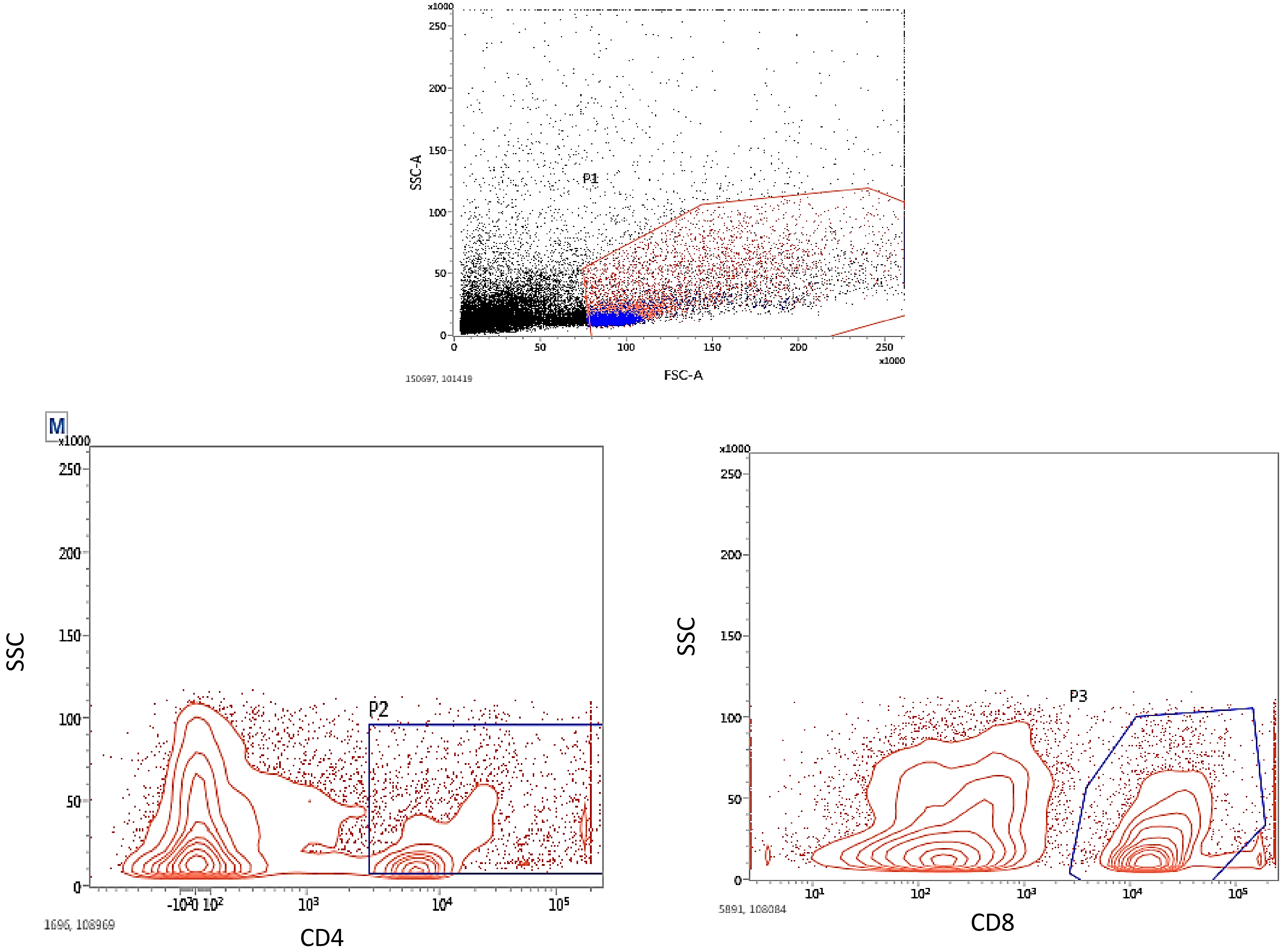

Supplementary Figure 3B

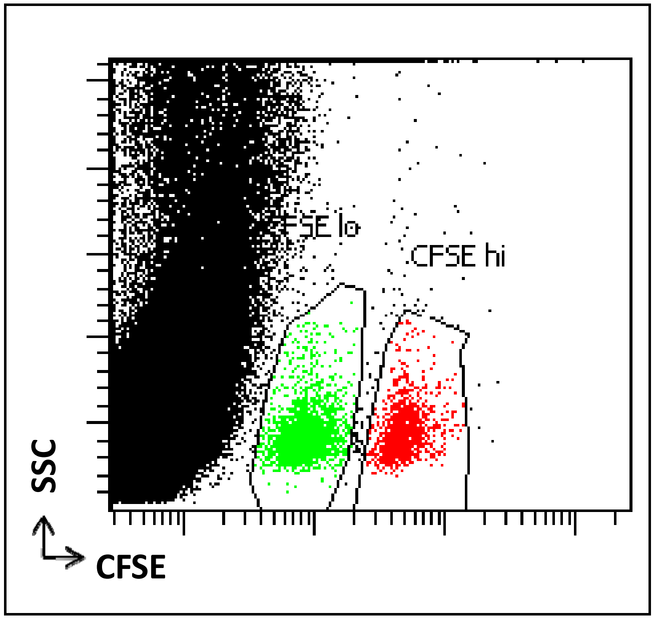

Supplementary Figure 4A: Comparison of BCG and BCG-L4.8

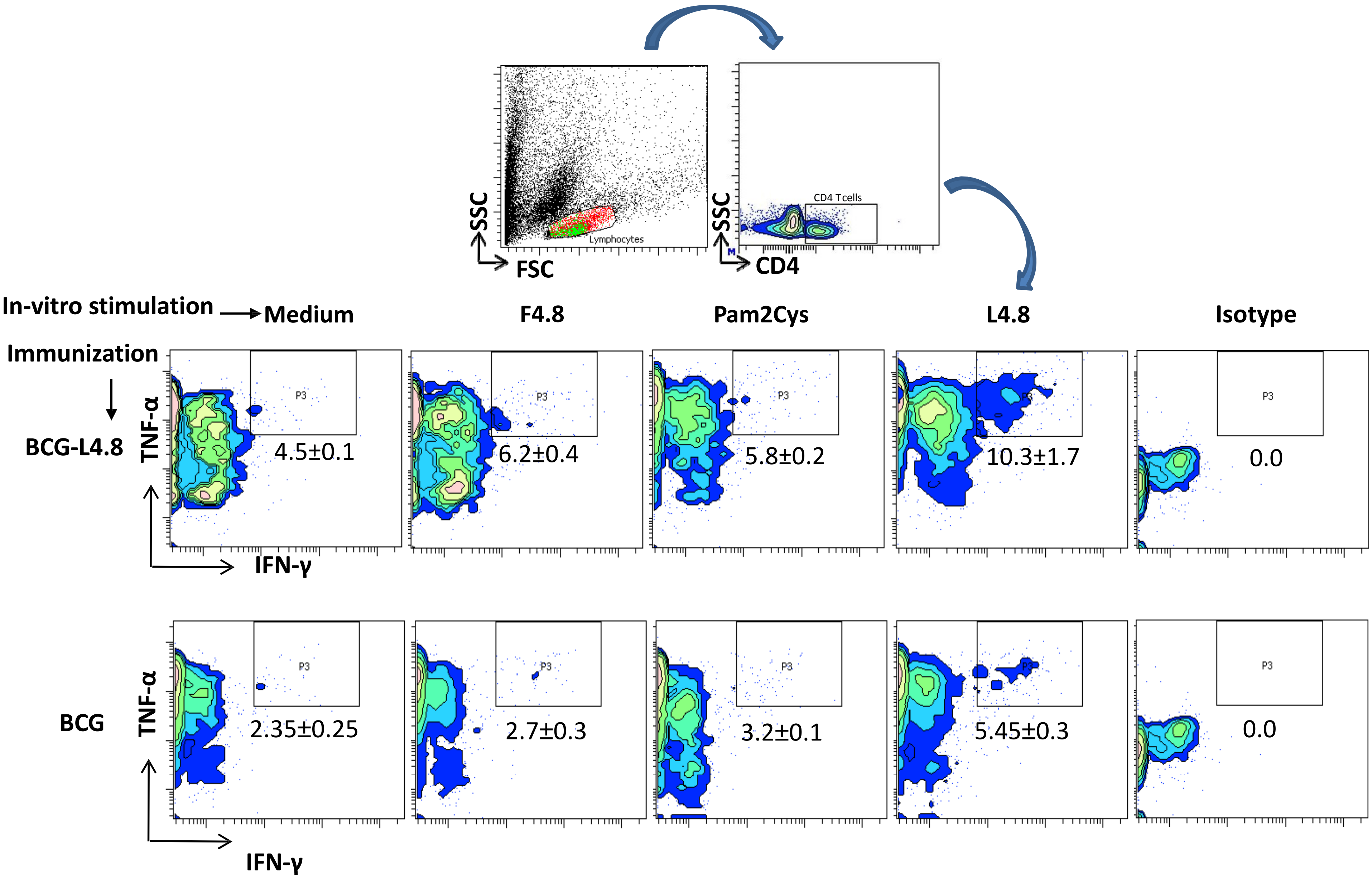

Supplementary Figure 4B: Comparison of BCG and BCG-L4.8

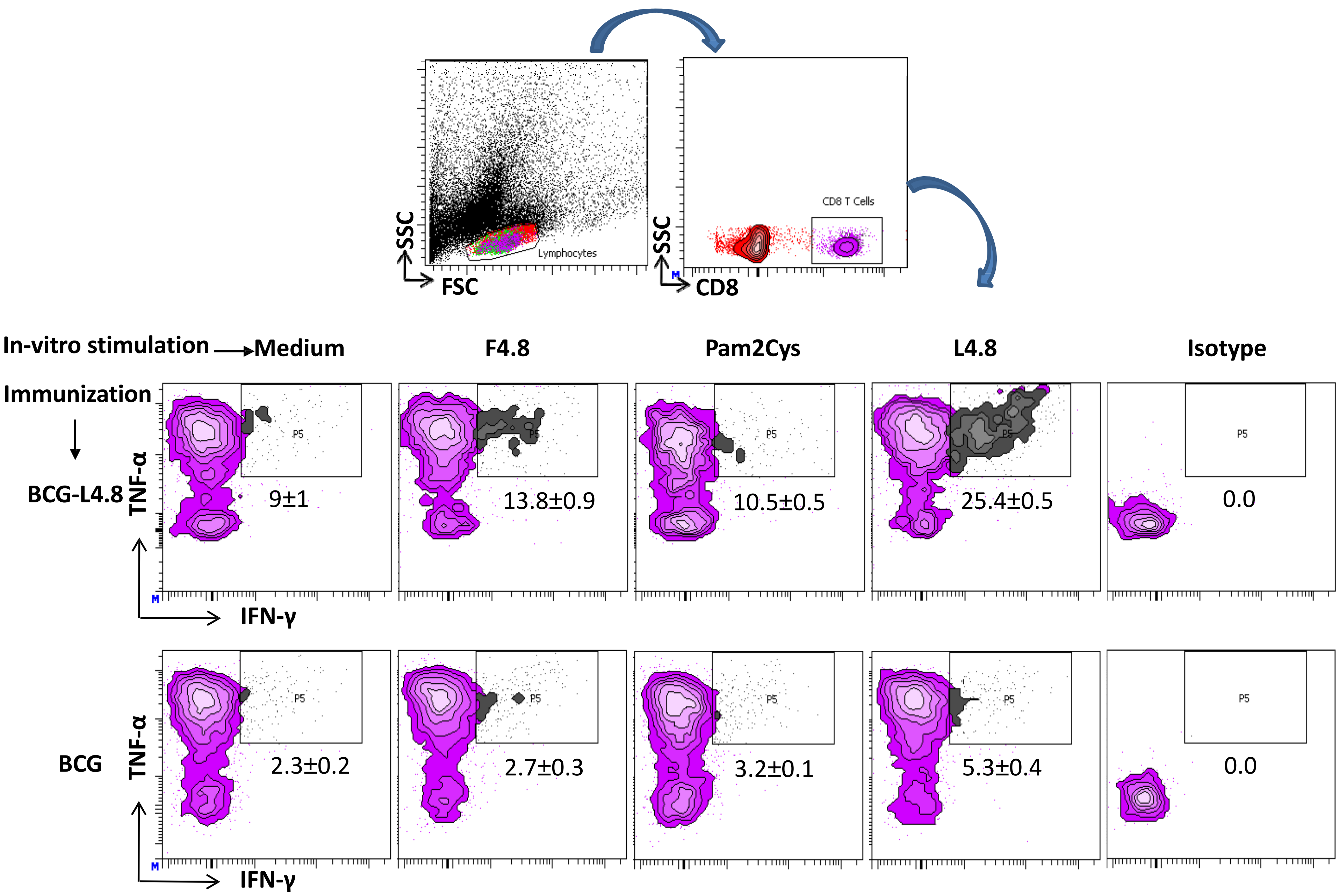

Supplementary Figure 4C: Comparison of BCG and BCG-L4.8

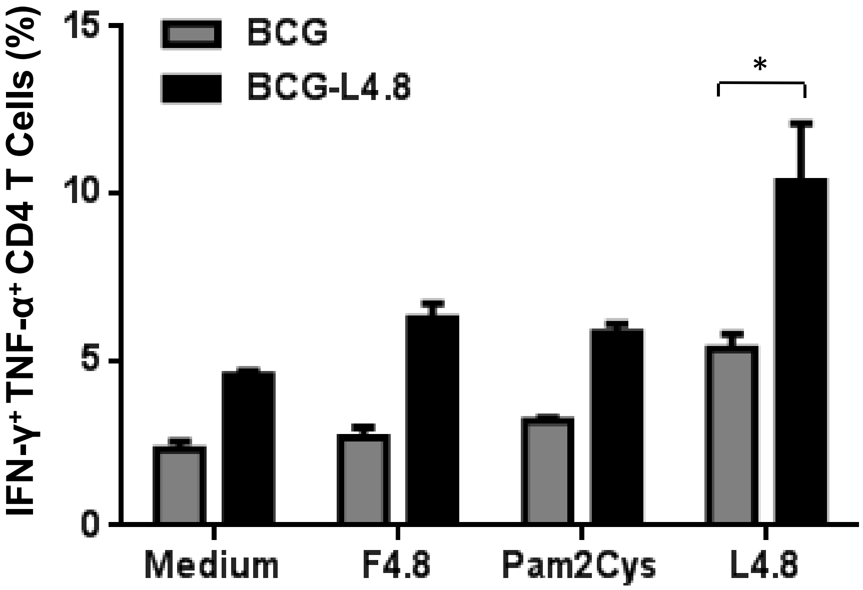

Supplementary Figure 4D: Comparison of BCG and BCG-L4.8

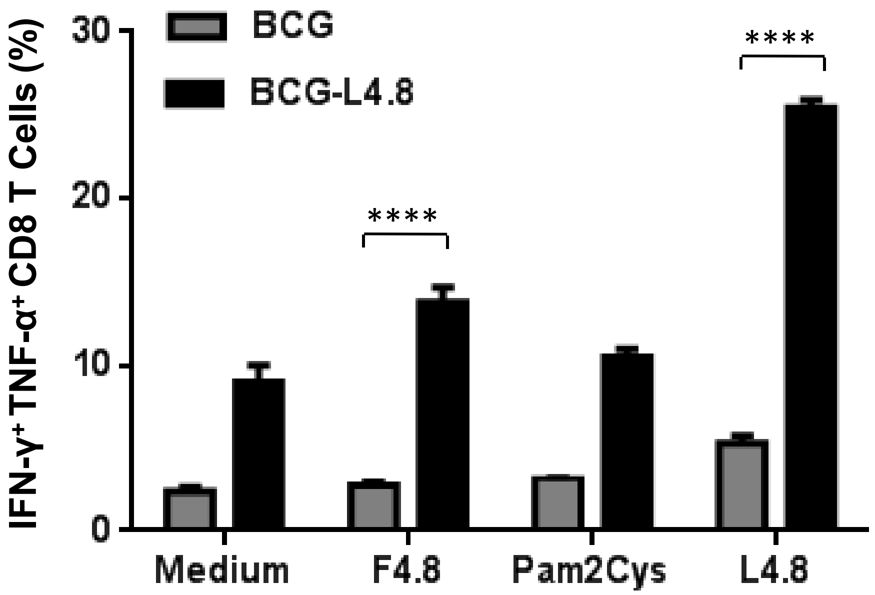

Supplement: Supplementary file 2 — Additional file 2: Figure S1. Titration of peptide dose for in vitro stimulation. Mice were immunized with L91 or L4.8 (20 nmol) and boosted after 7 d (10 nmol). Splenocytes were obtained after 15 d of immunization and in vitro stimulated with different concentrations of (A) L91 and (B) L4.8 for 72 h. Splenocyte proliferation was assessed by 3H-thymidine incorporation assay and expressed as counts per minute (cpm). Figure S2. Gating strategy for CD4+ T cells and CD8+ T cells. The P1 gate was made on lymphocytes, P2 gate on SSC-A/CD4+ T cells and P3 on SSC-A/CD8+ T cells zones. Further, analysis of T cells was done on the gated cells. Figure S3. Gating strategy for monitoring the expression of KLRG1+ T cells and target lysis. (A) The P1 gate was made on lymphocyte zone. P2 gate on SSC-A and CD4+ T cells while P3 gate on SSC-A and CD8+ T cells. (B) For target lysis two gates were made in CFSEhi and CFSElo cells on SSC-A. Later, histogram was plotted for the analysis. Figure S4. Gating strategy for monitoring the expression of IFN-γ+ and TNF-α+ CD4 T cells. (A, B) The primary gate was made on lymphocyte zone and secondary gate on SSC-A and (A) CD4+ T cells; (B) CD8 T cells. The percentage of IFN-γ+/TNF-α+ was monitored on secondary zones (CD4+/SSC-A; CD8+/SSC-A). The isotype-matched control did not show any double positive T cell population. (C, D) Bar diagram represents the comparison of polyfunctional (C) CD4 T cells and (D) CD8 T cells (IFN-γ+ TNF-α+) obtained from BCG and BCG-L4.8 groups. The data are represented as mean ± SEM. *p ≤ 0.05; ****p ≤ 0.0001. [file 12967_2018_1653_MOESM2_ESM.pdf]
